# Fecal Mycobiota in Patients with Inflammatory Bowel Diseases and Extraintestinal Manifestations

**DOI:** 10.1080/29933935.2024.2400071

**Published:** 2024-09-20

**Authors:** Sandra Hertz, Hans Linde Nielsen, Sabrina Just Kousgaard, Lone Larsen, Henrik Nielsen

**Affiliations:** a Department of Infectious Diseases, Aalborg University Hospital, Aalborg, Denmark; b Department of Clinical Medicine, Aalborg University, Aalborg, Denmark; c Department of Clinical Microbiology, Aalborg University Hospital, Aalborg, Denmark; d Department of Gastrointestinal Surgery, Aalborg University Hospital, Aalborg, Denmark; e Department of Gastroenterology and hepatology, Aalborg University Hospital, Aalborg, Denmark; f Center for Molecular Prediction of Inflammatory Bowel Disease, PREDICT, Department of Clinical Medicine, Aalborg University, Aalborg, Denmark

**Keywords:** Gut mycobiota, fungal microbiota, inflammatory bowel disease, extraintestinal manifestations, internal transcribed spacer sequencing (ITS sequencing), Oxford Nanopore

## Abstract

Inflammatory Bowel Disease (IBD), a chronic inflammatory condition affecting the gastrointestinal tract, is associated with extraintestinal manifestations (EIMs) in up to 50% of patients, significantly impacting quality of life and causing serious complications. Reduced gut microbiota bacterial diversity is a characteristic of IBD, and emerging evidence suggests fungal changes, such as an increase in *Candida*, are also present. However, the mycobiota in patients with IBD, particularly those with EIMs, remains sparsely investigated. Fecal samples from 107 patients with IBD and 43 healthy controls (HC) underwent Internal Transcribed Spacer 2 (ITS2) sequencing using Oxford Nanopore technology. Fecal mycobiota composition differed between patients with IBD and HC, with increased *Malassezia* in IBD. Patients with IBD and EIMs exhibited lower fungal richness compared to those without EIMs, however did not differ in beta diversity. Patients with IBD and primary sclerosing cholangitis (PSC, *n* = 7) possessed a distinct mycobiota composition compared with HC and IBD without EIMs, characterized by lower diversity and decreased relative abundance of *Saccharomyces cerevisiae* and unspecified *Dipodascaceae*. Overall, the fecal mycobiota of the whole study group (*n* = 150) exhibits heterogeneity and low richness, primarily shaped by the dominant genus. In conclusion, minor fecal mycobiota differences related to EIMs were observed, particularly in PSCs.

## Introduction

Inflammatory Bowel Disease (IBD) is a multifaceted disease characterized by significant clinical diversity related to severity, treatment response, and complications.^1–3^ Additionally, up to half of all patients with IBD will experience extraintestinal manifestations (EIMs), with a higher incidence in Crohn’s Disease (CD) compared to Ulcerative Colitis (UC).^4^ The current understanding of IBD etiology is multifactorial, involving genetic predisposition, alterations in the gut microbiota, immunological, and environmental factors.^5^ Alterations in the gut microbiota of patients with IBD are well characterized with a reduction of bacterial diversity and a loss of obligate anaerobic bacteria such as *Faecalibacterium prausnitzii*.^6,7^ While much of the gut microbiota research in IBD has focused on gut bacteria, it is important to recognize that the microbiota comprises a complex ecosystem of various microorganisms. Among these, the fungal community, referred to as the mycobiota, represents 0.1–2% of the total microbial community in the healthy human gut.^8^

A recent comprehensive study combining 16 cohorts (*n* = 3363) characterized the human gut mycobiota and identified four fungal enterotypes.^9^ These enterotypes correlated with bacterial enterotypes and were stable across different populations and geographical locations.^9^ The most abundant genera identified were *Candida, Saccharomyces, Penicillium* and *Aspergillus*, which are also abundant in other body sites.^10^

Gut bacteria can limit fungal colonization through immune activation and metabolite production.^8,11,12^ During intestinal inflammation, reduced bacterial diversity can occur, which creates a niche for fungal expansion.^8,13,14^ Recent studies have described altered fecal mycobiota in IBD, characterized by an increase in *Candida* and decrease in *Saccharomyces* as well as changes in the *Ascomycota/Basidiomycota* ratio.^8,15^ A limited number of studies have investigated mycobiota differences in relation to IBD phenotype, disease activity, and treatment response.^15^ A comprehensive review by *Balerramo et al*. presents the current knowledge and gaps in mycobiota research in IBD.^15^ In CD, the stricture phenotype (B2) was found to be associated with increased *Candida albicans* in both Norwegian^16^ and Chinese cohorts.^17^ Mycobiota differences related to Montreal classification of disease location (L) were also described in the Norwegian cohort. Specifically, CD patients with only ideal involvement (L1) were characterized by increased relative abundance of *Debaryomyces hansenii* and decreased *Candida tropicalis*.^16^ Another study described the enrichment of several fungal genera in patients with CD with perianal disease.^17^ Less is known about UC phenotypes, but *Catalán-Serra et al*. reported a decrease in *Penicillium* relative abundance to be associated with patients with pancolitis (E3).^16^ Mycobiota compositional changes have been linked to IBD flares and disease severity, including increased abundance of the ratio of *Basidiomycota* to *Ascomycota* ratio,^13,16^ increased *Candida* relative abundance,^13,16,18^ and conflicting findings for *Saccharomyces* and *Penicillium*.^13,17–19^ Finally, fungal composition has also been related to IBD treatment response, with non-responders to infliximab treatment having a higher abundance of *Candida*.^20^

Our research group and others have found a reduction of gut bacterial diversity related to the presence of EIMs.^21–25^ Reduced gut bacterial diversity is known to impact the bacterial inhibition of fungal colonization, creating a favorable environment for fungal proliferation.^8,13,14^ Despite this, studies on mycobiota in patients with IBD and EIMs are sparse, as highlighted by *Balderramo* et al..^15^ In this study, we aimed to characterize the mycobiota in patients with IBD compared with healthy controls (HC) as well as compare patients with IBD and a range of different EIMs to patients with IBD without EIMs.

## Methods

### Study population

The study consists of participants from the IBD-X study^25^ and healthy controls (HC). IBD-X participants were adult patients with IBD (CD: ICD-10: K50 or UC:ICD-10: K51) with or without EIMs and were included from a single center (Aalborg University Hospital, Denmark) between 2020–2022.^25^ Patients with IBD with or without EIMs were identified as described previously^25^ Exclusion criteria included systemic antimicrobial therapy within 30 days of sample collection, terminal illness, cognitive disorders such as dementia, pregnancy, or breastfeeding.^25^ HCs were recruited from the Blood Bank at Aalborg University Hospital, Aalborg, Denmark and through advertisement at the hospital’s website. The study was approved by The North Denmark Region Committee on Health Research Ethics (IBD-X: *N*-20190021, HC: 20180013) and all participants provided written consent before participation.

### Sample and data collection

All IBD-X participants and HCs provided a stool sample. Stool samples were collected at home as previously described.^25^ Additionally, IBD-X participants provided a blood sample within 24 h of stool sample collection. Blood analyses included C-reactive protein, hemoglobin, platelets, albumin, alkaline phosphatase, alanine transaminase, and bilirubin and leukocytes incl. neutrophils, lymphocytes, monocytes, eosinophils, and basophils.^25^ All IBD-X participants filled out a symptom questionnaire at study inclusion. The questionnaire included self-reported life quality and disease activity scores such as Short Health Score (SHS), Harvey Bradshaw Index (HBI), and Simple Colitis Clinical Activity Index (SCCAI).^25^ Additional clinical data related to their IBD and EIM diagnosis were collected through medical records.^25^ Age, sex, and BMI were noted for HCs.

### Fungal internal transcribed spacer 2 (ITS2) sequencing

DNASense in Aalborg, Denmark, performed DNA extraction and ITS2 sequencing. DNA was extracted using a revised DNeasy 96 PowerSoil Pro QIAcube HT Kit method.^26^ DNA extracts were quantified and used to amplify the Internal transcribed spacer region 2 (ITS2) region by PCR using the following program: Initial denaturation at 98°C for 3 min, 25 cycles of amplification (98°C for 30 s, 62°C for 20 s, 72°C for 2 min) and a final elongation at 72°C for 7 min. The forward and reverse primers used include custom 24 nt barcode sequences followed by the sequences targeting ITS2 region: [ITS2–9] TACACACCGCCCGTCG and [ITS4] TCCTSCGCTTATTGATATGC.^27^ Amplicon libraries were purified using the standard protocol for CleanNGS SPRI beads (CleanNA, NL). DNA was eluted in 25 μL of nuclease free water (Qiagen, Germany). Sequencing libraries were prepared from the purified amplicon libraries using the SQK-LSK114 kit (Oxford Nanopore Technologies, UK) according to manufacturer protocol with the following modifications: 500 ng total DNA was used as input, and CleanNGS SPRI beads for library clean-up steps. DNA concentration was measured using Qubit dsDNA HS Assay kit (Thermo Fisher Scientific, USA). Gel electrophoresis using Tapestation 2200 and D1000/High sensitivity D1000 screentapes (Agilent, USA) was used to validate product size and purity of a subset of amplicon libraries. The resulting sequencing library was loaded onto a PromethION R10.4.1 flowcell and sequenced using the MinKNOW 23.11.4 software (Oxford Nanopore Technologies, UK). Reads were basecalled and demultiplexed with MinKNOW Dorado 7.2.13 using the super accurate basecalling algorithm (config r10.4.1_400bps_sup.cfg) and custom barcodes.

### Bioinformatic processing

The sequencing reads in the demultiplexed and basecalled fastq files were filtered for length (320 2000 bp) and quality (phred score > 15) using a local implementation of filtlong v0.2.1 with the settings – min_length 320 – max_length 2000 –min_mean_q 97. The filtered reads were mapped to the QIIMEformatted and 99% identity clustered UNITE database release 9.0^28^ with minimap2 v2.24r1122 using the axmapont command^29^ and downstream processing using samtools v1.14.^30^ Potential generic placeholders and dead-end taxonomic entries were cleared from the taxonomy flat file, i.e., entries containing uncultured, metagenome or unassigned, were replaced with a blank entry. Mapping results were filtered such that alignment length was >125 bp and mapping quality >0.75. Low-frequency OTUs detected in <0.01% of the total mapped reads within each sample were removed as a data denoising step. Samples with <1000 mapped DNA reads were also removed from the dataset. Additionally, a single OTU of *Thelephora* was identified as a contaminant and removed. Further bioinformatic processing was done using R version 4.3.2 (2023-10-31) and using the R packages: ampvis2 (2.8.7),^31^ tidyverse (2.0.0), seqinr (4.2.36), ShortRead (1.60.0) and iNEXT (3.0.0).^32,33^

### Statistical analysis

Statistical analyses were conducted using R (v4.2.2) or GraphPad Prism (v9.5.0). Mycobiota was compared between IBD participants (*n* = 107) and HC (*n* = 43), between IBD subtype CD (*n* = 56) and UC (*n* = 41) as well as between patients with IBD (IBD-EIM, *n* = 64) with or without EIMs (IBD-C, *n* = 33). In addition, EIMs were compared for CD [CD-EIM (*n* = 42) vs CD-C (*n* = 14)] and UC [UC-EIM (*n* = 21) vs UC-C (*n* = 19)] separately. Data distribution of parametric data was evaluated for each variable applying t-test or one-way ANOVA for normally distributed data and Mann Whitney (MW) or Kruskal Wallis (KW) for non-normally distributed data. Categorical data were analyzed using the Chi-square (χ^2^) or Fisher’s exact test (FE) when applicable. ITS2 alpha diversity indices (such as richness) were calculated through “phyloseq” in R (v1.40.0) and “vegan” (v2.6.4).^34,35^ Distance matrices were calculated using “phyloseq” and related to clinical variables using Permutational Analysis of Variance test (PERMANOVA) using “adonis2” of the “vegan” package (v2.6.4)^35^ to relate mycobiota composition (beta-diversity) to clinical variables. Differential abundant OTUs were identified using DESeq2 (Differential expression analysis based on the Negative Binomial distribution) in R.^36^ OTUs present in more than 10% of samples with False discovery rate – corrected p-values (p.fdr) <.05, and log fold changes >1 or <-1 were considered significant.

## Results

### Clinical characteristics of study participants

There was no significant difference in age and sex between IBD- and healthy participants, however patients with IBD had a higher BMI compared to HC (MW: *p* = .02) (Table 1). Significant differences in clinical variables between patients with IBD with and without EIMs in the IBD-X study population have previously been described.^25^ In short, IBD-EIM participants were characterized by higher BMI, greater history of IBD-related admission, decreased quality of life (Short Health Scale, SHS), increased self-reported disease activity scores (HBI/SCCAI), higher fecal calprotectin levels and greater use of biological treatments such as TNFα-inhibitors.^25^

**Table 1.** Age, sex and BMI of IBD-X (*n* = 107) and healthy controls HC (*n* = 43).

	IBD (*n* = 107)	HC (*n* = 43)
Age (mean (±SD))	47 (±13)	47 (±13)
Sex (female %)	58/107 (54%)	23/43 (54%)
BMI (mean (±SD))*	27,1 (±5,5)	24,8 (±3,3)

*p-value <0.05. Age and BMI were tested with a Mann–Whitney test; Sex was tested with a Fisher’s exact test.

### Overall fungal community description

**Figure 1.**
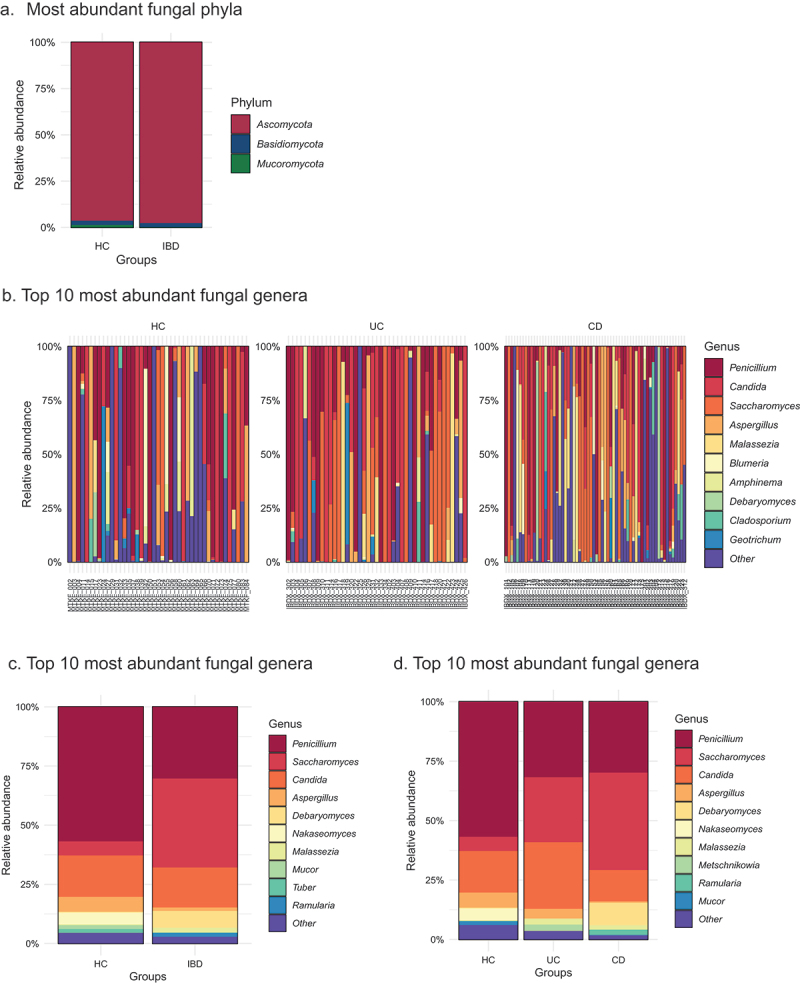
Fungal taxa distribution between groups.

**Figure 2.**
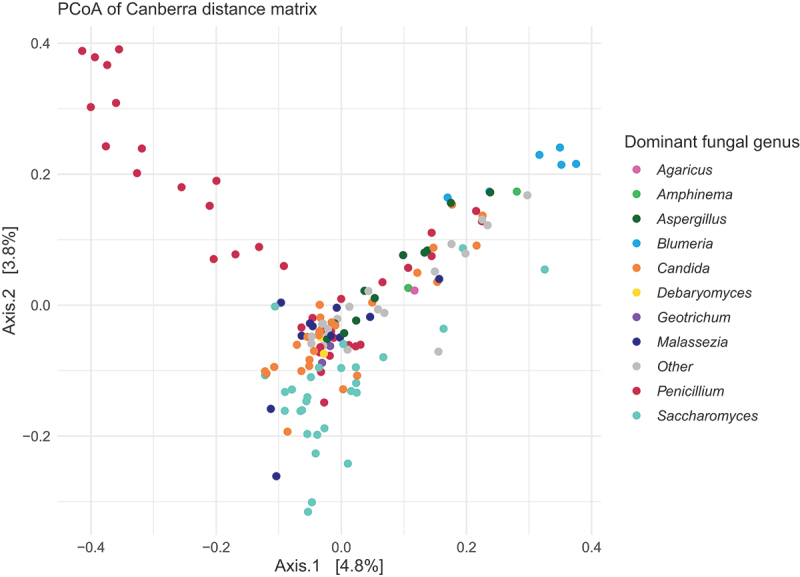
Principal coordinates analysis plot of fungal community.

A total of 150 of 179 samples passed quality filtering, 107 from IBD-X and 43 healthy subjects. Of the IBD samples, 81% had >1000 mapped DNA reads, whereas HC samples had >1000 reads in 96% of samples, which was significantly higher compared to the IBD group (FE: *p* = .02). A total of 269 fungal OTUs were identified in the study population, representing three fungal phyla (*Ascomycota*, *Basidiomycota* and *Mucoromycota*), comprising 17 classes, 67 families, and 88 genera and 144 species. The mean richness per patient was 7 (SD ± 5), emphasizing the lower richness of the fecal fungal community compared to bacteria.^25^ The top genera represented included, *Aspergillus, Candida, Debaryomyces, Malassezia, Penicillium* and *Saccharomyces*, all previously described as members of the human mycobiota.^9^ The mycobiota of all samples and clinical groups are illustrated in Figs. 1(A–D). The majority of detected fungi belonged to the *Ascomycota* (>90%; Fig. 1(A)). In general, fecal mycobiota exhibited a high degree of heterogeneity across the participants with only ten genera and nine species consistently detected in >10% of samples (Fig. 1(B)). For each participant, we identified the dominant fungal family and genus. Based on the dominant genus, 43% of participants (35% HC, 33% IBD) could be classified as one of the four previously described mycobiota enterotypes, *Candida*, *Saccharomyces*, *Aspergillus* and unspecified *Ascomycota*.^9^ In fact, the dominant genus emerges as the major determinant of variation in gut mycobiota beta-diversity (Fig. 2), illustrating sample clusters based on the dominant genus.

(A)Mycobiota (at phylum level) between IBD (*n* = 107) and HC (*n* = 43). (B) Mycobiota (genus level) for all samples divided into HC, UC, and CD groups. (C) Mycobiota (at genus level) between IBD and HC. (D) Mycobiota (at genus level) between CD, UC, and HC. Colored and labeled by top ten most abundant genera, the remaining low frequent/low abundant OTUs were categorized as “other”.

Canberra distance matrix. The fecal mycobiota of participants is colored by 10 most abundant dominant fungal genera, the remaining dominant fungal genera are labelled as ”Other”.

### Comparison between patients with IBD and healthy controls

There was no significant difference in fecal mycobiota alpha diversity between patients with IBD and HC (Richness; MW: *p* = .052; Pielou’s evenness; MW: *p* = .37; Shannon’s diversity; MW: *p* = .96). When comparing IBD subtypes, neither CD nor UC exhibited significantly different alpha diversity compared to HC. However, mycobiota composition (beta-diversity) measured using a Canberra distance matrix was significantly different between IBD and HC (PERMANOVA; R^2^ = 0.008, *p* = .02, supplemental Table 1) although the variance explained by this variable was small. Upon dividing the IBD group into its subtypes (CD and UC) the difference in composition reached higher significance (PERMANOVA; R^2^ = 0.018, *p* = .009, supplemental table S1). Age and sex, were not significant explanatory factors for fecal mycobiota composition (PERMANOVA; *p* > .05), however BMI as a categorical variable (underweight, normal, overweight, and obese) was significantly related to fecal mycobiota variance (R^2^ = 0.03, *p* = .027, suppl. table S1). At the genus level, differences between IBD and HC were observed (Fig. 1(C,D)). There was a higher detection of *Malassezia* in IBD patients (25%) vs HC (5%; FE: *p* = 0.004) and a trend toward a greater detection of *Saccharomyces* in IBD patients (44%) compared to HC (28%; FE: *p* = 0.06), and relative abundance of one OTU belonging to *Malassezia restricta* was increased in IBD (DESeq2, Log2FC: 27, p.fdr <.01), which was still significant after adjusting for BMI (DESeq2, Log2FC: 29. *p* < .01).

### Comparison between patients with UC and CD

There was no difference in alpha diversity between CD (*n* = 56) and UC (*n* = 41). However, there was a trend toward a significant difference in fungal composition between patients with CD and UC (Canberra: PERMANOVA: R^2^ = 0.011, *p* = .08, suppl.table S1). Genus level differences between CD and UC are depicted in Fig. 1(C). *Debaryomyces* detection was significantly higher in patients with CD (14%), compared to UC (2%; FE: *p* = .04), however there was no significant difference in the relative abundance in OTUs between CD and UC.

### Mycobiota differs in IBD patients with or without EIMs

**Figure 3.**
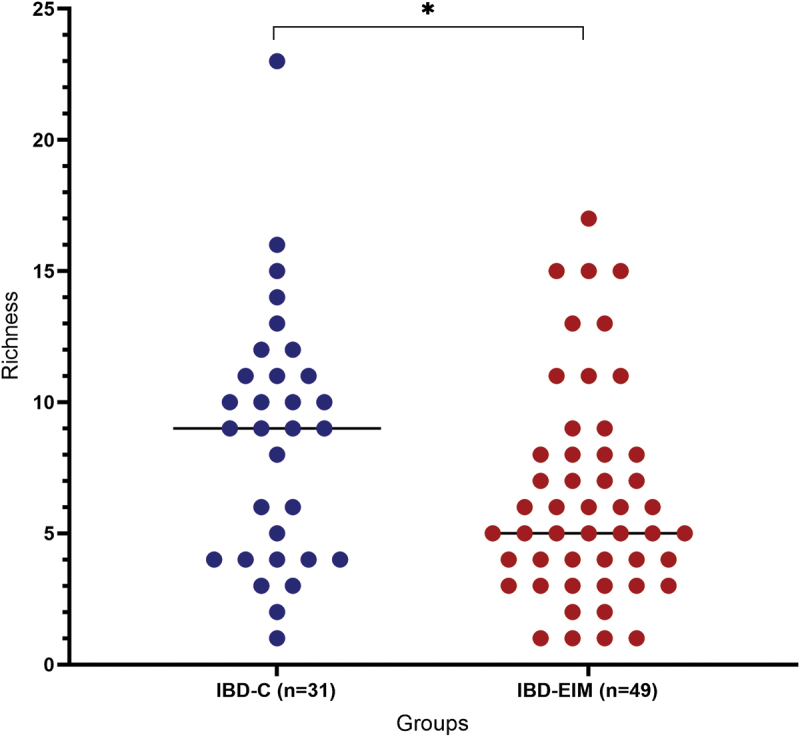
Fungal alpha diversity.

**Figure 4.**
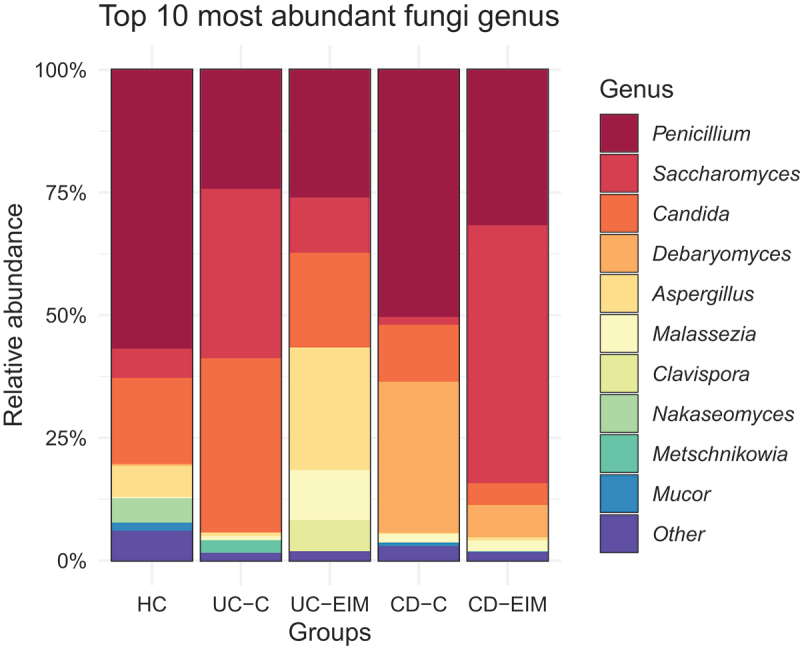
Fungal taxa distribution between patients with IBD with or without EIMs divided by IBD subtype.

Fecal mycobiota changes related to EIMs were investigated in IBD participants with a history of EIMs (IBD-EIM: *n* = 60) or no history of EIMs (IBD-C: *n* = 31). In total, 49/60 had EIMs at time of sample collection. IBD patients with EIMs at sample collection had a lower fungal richness compared to those without (MW: *p* = .04; Fig. 3). This was also evident when analyzing all with a history of EIMs (*n* = 60; MW: *p* = .01); however, no difference was found in other alpha diversity measures. IBD subtype analysis revealed that mycobiota richness was significantly decreased in UC-EIM compared with UC-C (MW; *p* = .03) and trending toward decreased richness in CD-EIM compared to CD-C (MW; *p* = .09). Overall history of EIMs and EIMs at sample collection did not explain variance in fecal mycobiota composition (Canberra, PERMANOVA, *p* > .05, suppl. table S1). Fungal relative abundances between CD-EIM vs CD-C, and UC-EIM vs UC-C are depicted in Fig. 4. Here, *Aspergillus* and *Malassezia* appeared higher in UC-EIM than UC-C, whereas *Debaryomyces* appeared higher in CD-C than CD-EIM, and *Saccharomyces* higher in CD-EIM in CD-C. However, no OTU was significantly different between IBD-EIM and IBD-C, even after adjusting for f-calprotectin, but one OTU belonging to *Dipodascaceae* was trending toward a lower relative abundance in IBD-EIM (DESeq: log2fc:-10, p.fdr = .06).

Richness between Inflammatory Bowel Disease patients with (IBD-EIM) or without (IBD-C) extraintestinal manifestations (EIM), *p* = 0.04.

CD-EIM (*n* = 42), CD-C (*n* = 13), UC-EIM (*n* = 18), and UC-C (*n* = 18), HC (*n* = 43).

To further investigate the difference in richness observed in patients with IBD and EIM, subanalysis of separate EIMs was performed. Only patients with IBD and primary sclerosing cholangitis (PSC) (*n* = 7) exhibited significantly decreased richness (MW, *p* = .04) and diversity (Shannon’s diversity: MW, *p* = .007) compared patients with IBD without PSC or any other EIM. Additionally, PSC explained a significant, although small portion of variance in fecal mycobiota composition (R^2^ = 0.014, *p* = .002, suppl. table S1). Here, *Saccharomyces* detection was significantly lower in IBD-PSC (0%) compared to IBD-C (48%) and HC (29%) (FE: *p* = .03). Since fungal community was significantly different for IBD-PSC, a comparative differential abundance analysis was performed to identify fungal taxa of importance. Six fungal OTUs were significantly decreased in PSC compared to HC (p.fdr <.05) and included an unspecified *Ascomycota*, *Saccharomyces cerevisiae*, *Aspergillus piperis,* and three unspecified *Dipodascaceae*. Five fungal OTUs were significantly decreased in IBD-PSC compared to IBD-C and included an unspecified *Ascomycota*, *Saccharomyces cerevisiae*, *Malassezia restricta* and two unspecified *Dipodascaceae*.

### Other IBD characteristics related to mycobiota composition

Various IBD-related variables were also investigated in relation to fecal mycobiota composition. PERMANOVA of Canberra distance matrices identified three additional clinical variables significantly explaining fungal community variance: 1) IBD surgery (R^2^ = 0.012, *p* = .037), including ileocecal resection (R^2^ = 0.012, *p* = .029) and colectomy (R^2^ = 0.012, *p* = .029), 2) current fistula (R^2^ = 0.022, *p* = .016), and 3) current GI abscesses (R^2^ = 0.031, *p* = .025). All PERMANOVA results are listed in suppl. table S1. IBD subtype analysis revealed additional significant clinical variables related to fungal composition in CD and UC, respectively (suppl. table S1). For patients with CD (*n* = 64) clinical variables such as total Short Health Score, IBD surgery (ileocecal resection), infliximab treatment, and osteopenia/osteoporosis were significantly explaining fungal composition variance (Canberra distance matrices). For patients with UC (*n* = 43) only colectomy with ileoanal-anastomosis was significant.

## Discussion

In this study, we present fecal mycobiota characteristics in patients with IBD compared to HC together with an in-depth analysis of clinical variables within IBD subgroups, such as EIMs providing novel insight on the mycobiota in IBD. In general, fungal detection and richness were low in both IBD and HC. Despite this, we observed significant differences between IBD and HC. Within the IBD cohort, we also observed minor differences dependent on IBD subtypes and EIMs, especially PSC.

Four different fungal enterotypes based on dominant fungi were recently described in a large multicohort study,^9^ which included: *Candida* (Can_type), *Saccharomyces* (Sacc_type), *Aspergillus* (Asp_type) and an unspecified *Ascomycota* (Asc_type). Can_type were associated with older age and certain diseases, including type 2 diabetes, alcoholic hepatitis, *Clostridioides difficile* infection, and Alzheimer’s disease, after adjusting for age.^9^ In our study, we observed that 43% of participants (35% HC, 33% IBD) could be classified under one of the four enterotypes,^9^ indicating that other enterotypes exist. Additional studies of more cohorts may help to describe the complex picture of enterotypes.

Fungal richness and diversity between IBD and HC groups did not differ, however a small compositional difference was observed. Previous studies have described an altered fecal mycobiota in IBD characterized by increase of *Candida* and decrease of *Saccharomyces* and a shift in the *Ascomycota* to *Basidiomycota* ratio.^8,13,15^ In contrast to these findings, we did not observe differences in *Candida*, though we did observe a trend towards an increase in *Saccharomyces* in the IBD group.

Fungal involvement in the pathogenesis of IBD has been suspected for some time as *anti-Saccharomyces cerevisiae antibodies* (ASCA) have been found to be increased especially in patients with CD and have gained interest as a biomarker for CD.^37^ Despite the name, ASCA is not species specific to *Saccharomyces cerevisiae*, but directed toward mannan, a common component of fungal cell walls.^37^ *Malassezia* was higher in IBD compared to HC. *Malassezia* can be found on the skin and has been associated with dermatological conditions such as pityriasis versicolor, atopic eczema, and seborrheic dermatitis.^38^ The presence of *Malassezia* in the human gut has been described previously^39,40^ and has also been linked to IBD, where *Malassezia restricta* has been found in higher abundances in colonic mucosa of CD patients and associates with CARD9 risk alleles.^41^ Additionally, *Malassezia* causes a stronger pro-inflammatory response than the larger yeast *C. albicans* and *S. cerevisiae*, can be recognized by ASCA, and exacerbates colitis in a mouse model.^41^ *Debaryomyces* detection were higher in CD compared to UC. Interestingly, *Debaryomyces* is commonly found in dietary components, e.g., cheese^8^ and has previously been found to be increased in patients with CD as well as implicated in impaired gut healing in a CD mouse model.^42^ Furthermore, *Debaryomyces* has been found increased in patients with UC compared to healthy controls and was associated with more severe disease.^43^

Our study group has previously described a decrease of bacterial diversity in patients with EIM compared to those without,^25^ and here we report a further decrease in fungal richness, although this did not affect the overall fungal composition to a degree that was statistically significant. In fact, it was only the EIM of PSC, which was significantly different, suggesting that fungi might only be implicated in specific types of EIMs. PSC is a chronic fibrotic disease of the bile ducts causing cirrhosis and ultimately liver failure.^44^ Approximately 60–80% of patients with PSC have IBD, most commonly UC, which is why it is considered an EIM of IBD.^44^ PSC diagnosis was the most significant clinical variable that explained mycobiota variance. Although we recognize that this group of patients was small (*n* = 7), significance was sustained in a sub-analysis of UC patients, which was the IBD diagnosis for most patients with PSC. IBD-PSC patients exhibited significantly lower fungal alpha diversity compared with either HC subjects or patients with IBD without PSC and were compositionally distinct with decrease/loss of several fungal taxa such as *Saccharomyces cerevisiae* and *Dipodoscacae*. The fungal antibody ASCA has previously been described as highly prevalent in patients with PSC *(53%)* .^45^ Furthermore, three studies of the fecal mycobiota in patients with PSC have been previously described, but with different findings. One study observed higher fungal diversity in patients with either PSC or PSC with IBD compared to patients with IBD without PSC with an increase in *Exophiala* and *Sordariomycetes* class and decrease of *Saccharomyces cerevisiae*.^46^ Another found no difference in diversity between patients with PSC, healthy individuals and IBD, but demonstrated an increase in *Candida* and *Sordariomycetes*.^47^ Finally, investigating the mycobiota in pediatric patients with UC and UC-PSC revealed no difference in alpha diversity between groups but found increased *Saccharomyces and Debaryomcyces* relative abundance in UC-PSC.^48^ Increase of *Saccharomyces* in UC-PSC^48^ is in contrast to our findings and those of Lemoinne^46^ and *Rühlemann*.^47^ Differences in mycobiota findings between studies might be a result of cohort variation (e.g. adults vs. pediatrics), as all three studies targeted ITS2 and were sequenced on Illumina MiSeq. A multicenter and multinational study of mycobiota could clarify if general mycobiota changes are related to PSC.

### Limitations

First, the origin of the fungi detected in fecal samples is unknown and could originate from colonization of the gut and/or represent transient ingested fungi. Since we employed a DNA-based biomarker sequencing approach, the viability of these species and their functional effect on the host remains unknown. However, constant exposure of transient fungi has been reported to affect the immune system.^8^ Additionally, gut microbiota perturbations, as observed in IBD, are thought to provide a favorable niche for transient fungi to colonize the gut mucosa^8^

Second, the choice of gene and sequencing method can affect findings, and previous studies have used short-read sequencing such as Illumina, whereas we have used long-read-sequencing by Oxford Nanopore Technology (ONT), which can challenge comparison with previous studies. Short-read sequencing methods dominate marker gene-based microbiota studies due to its low error rate <0.1%. Previously, long-read sequencing was prone to higher error rates than short-read methods,^27^ but in 2021, a substantial technological improvement occurred with the R10 pore, which could improve accuracy to >99%.^49^ Recent methodological studies comparing the two methods have reported that ONT is suitable for gene amplicon sequencing,^50–52^ however its application in the field is still low. Long-read sequencing was first attempted on fungal communities in 2021,^53^ and to our knowledge it has not been used for gut mycobiota studies before. In addition, the gene choice (ITS1 vs ITS2) can also affect findings.^54^ ITS1 gene is more variable resulting in greater taxonomic resolution, but variable length and intron frequency can lead to inaccurate taxonomic identification.^54^ ITS2 is a more conserved gene with universal primers and higher PCR success rates, but with reduced taxonomic resolution compared to ITS1.^54^

Third, we observed significant findings in patients with IBD and PSC. However, the small sample size raises the possibility of this finding being random. In particular, the loss of *Saccharomyces* in PSC could be incidental, given that not all IBD-C or HC were positive for *Saccharomyces*. This emphasizes the need to verify these fungal findings in larger cohorts and interpret this finding with caution. Additionally, ursodeoxycholic acid (UDCA) is commonly used in PSC management, thus it could be speculated that the distinct fecal mycobiota exhibited by these patients is related to UDCA treatment, however none of the seven PSC patients were being treated with UDCA at the time of sample collection.

## Conclusion

The fecal mycobiota of the whole study group (*n* = 150) exhibits heterogeneity and low richness, primarily shaped by the dominant genus. The mycobiota was related to IBD characteristics such as history of IBD-related surgery and perianal disease. Patients with IBD have a different mycobiota composition characterized by an increase of *Malassezia* compared to HC. Within IBD, *Debaryomyces* detection was greater in CD compared to UC. Overall, only minor fecal mycobiota changes, such as reduced richness, was related to the presence of extra-intestinal manifestations in IBD. However, patients with IBD and PSC appear to have a distinct mycobiota composition, although represented by a small sample size, this finding calls for further research into mycobiota in patients with PSC.

## Supplementary Material

Supplemental table 1.docx

## Data Availability

Mycobiome sequencing data are deposited in NCBI’s Sequencing Read Archive (accession number: PRJNA1148911 (https://www.ncbi.nlm.nih.gov/sra/PRJNA1148911)). The data supporting the findings of this study are available from the corresponding author [S.H.] upon reasonable request.
