# Supplementary material for: Fecal Mycobiota in Patients with Inflammatory Bowel Diseases and Extraintestinal Manifestations
Source: Gut Microbes Rep. 2024 Sep 20;1(1):2400071. doi: 10.1080/29933935.2024.2400071 (PMC12940096; doi:10.1080/29933935.2024.2400071)
Supplement: Supplemental table 1.docx [file KGMR_A_2400071_SM6053.docx]

| Supplemental table 1: PERMANOVA Canberra DM | | | | | | | | |
| --- | --- | --- | --- | --- | --- | --- | --- | --- |
|  | **Total study group**  **(n=150)** | | **IBD**  **(n=107/91)** | | **CD**  **(n=64)** | | **UC**  **(n=43)** | |
|  | **R^2^** | **P** | **R^2^** | **P** | **R^2^** | **P** | **R^2^** | **P** |
| domfungalGenus | **0.278** | **0.001** | **0.260** | **0.001** | **0.335** | **0.001** | **0.247** | **0.002** |
| domfungalFamily | **0.230** | **0.001** | **0.216** | **0.001** | **0.291** | **0.001** | **0.209** | **0.001** |
| Age | 0.005 | 0.973 | 0.007 | 0.942 | 0.013 | 0.923 | 0.019 | 0.952 |
| Sex | 0.007 | 0.102 | 0.010 | 0.199 | 0.014 | 0.725 | 0.026 | 0.187 |
| BMI (category) | **0.030** | **0.027** | 0.030 | 0.161 | 0.050 | 0.174 | 0.046 | 0.598 |
| BMI | 0.007 | 0.1 | 0.010 | 0.254 | 0.016 | 0.393 | 0.024 | 0.431 |
| HC vs IBD | **0.008** | **0.021** |  |  |  |  |  |  |
| HC vs CD vs UC | **0.016** | **0.008** |  |  |  |  |  |  |
| IBD type (CD vs UC) |  |  | **0.011** | **0.08** |  |  |  |  |
| PSC |  |  | **0.013** | **0.002** | 0.016 | 0.398 | **0.035** | **0.004** |
| EIM (yes/no) |  |  | 0.020 | 0.092 | 0.032 | 0.294 | 0.050 | 0.238 |
| EIMcount (0,1,≥2) |  |  | 0.017 | 0.779 | 0.029 | 0.755 | 0.043 | 0.833 |
| EIMatincl |  |  | 0.011 | 0.16 | 0.017 | 0.166 | 0.026 | 0.206 |
| Yearssincediagnosis |  |  | 0.008 | 0.64 | 0.013 | 0.922 | 0.024 | 0.329 |
| A_ageonset |  |  | 0.059 | 0.143 | 0.101 | 0.077 | 0.096 | 0.42 |
| SHS |  |  | 0.009 | 0.491 | 0.019 | 0.046 | 0.021 | 0.72 |
| AIH |  |  | 0.009 | 0.51 | 0.015 | 0.41 | 0.024 | 0.178 |
| Spa/Joint pain unspecified | |  | 0.008 | 0.664 | 0.017 | 0.199 | 0.018 | 0.987 |
| ArthralgiaNow |  |  | 0.010 | 0.298 | 0.016 | 0.408 | 0.024 | 0.41 |
| Uveitis |  |  | 0.018 | 0.53 | 0.030 | 0.601 | 0.021 | 0.743 |
| Pyoderma gangrenosum |  |  | 0.018 | 0.787 | 0.030 | 0.748 | NA | NA |
| Erythema nodusom |  |  | 0.018 | 0.647 | 0.030 | 0.743 | NA | NA |
| Thrombosis |  |  | 0.007 | 0.994 | 0.013 | 0.991 | 0.024 | 0.55 |
| Urolithiasis |  |  | 0.008 | 0.72 | 0.012 | 0.952 | 0.026 | 0.172 |
| Cholelithiasis |  |  | 0.009 | 0.46 | 0.013 | 0.839 | 0.026 | 0.208 |
| Psoriasis |  |  | 0.010 | 0.115 | 0.015 | 0.43 | 0.022 | 0.665 |
| Bone density disorder |  |  | 0.011 | 0.103 | **0.020** | **0.029** | 0.023 | 0.553 |
| Fistula (ever) |  |  | 0.010 | 0.196 | 0.015 | 0.463 | 0.025 | 0.039 |
| Fistula (current) |  |  | **0.022** | **0.016** | 0.034 | 0.075 | NA | NA |
| Absces (ever) |  |  | 0.010 | 0.084 | 0.017 | 0.152 | 0.025 | 0.056 |
| Absces (current) |  |  | **0.031** | **0.025** | 0.050 | 0.077 | NA | NA |
| Fissura (ever) |  |  | 0.010 | 0.235 | 0.016 | 0.425 | 0.025 | 0.167 |
| Fissura (current) |  |  | 0.020 | 0.254 | 0.033 | 0.654 | NA | NA |
| dominant bacterial Genus | |  | 0.152 | 0.316 | 0.180 | 0.103 | 0.240 | 0.321 |
| dominant bacterial Family | |  | 0.076 | 0.288 | 0.111 | 0.407 | 0.092 | 0.696 |
| F-calprotectin |  |  | 0.008 | 0.713 | 0.014 | 0.772 | 0.025 | 0.292 |
| Leukocytes |  |  | 0.010 | 0.227 | 0.017 | 0.192 | 0.023 | 0.55 |
| Neutrofiles |  |  | 0.010 | 0.311 | 0.018 | 0.159 | 0.024 | 0.609 |
| Lymphocytes |  |  | 0.009 | 0.502 | 0.014 | 0.817 | 0.026 | 0.259 |
| Monocytes |  |  | 0.010 | 0.33 | 0.017 | 0.288 | 0.026 | 0.257 |
| Albumin |  |  | 0.009 | 0.345 | 0.014 | 0.673 | 0.022 | 0.611 |
| IBD Family disposition |  |  | 0.009 | 0.438 | 0.016 | 0.491 | 0.022 | 0.752 |
| Standard treatment |  |  | 0.009 | 0.539 | 0.015 | 0.585 | 0.019 | 0.939 |
| Biological_treatment |  |  | 0.008 | 0.86 | 0.016 | 0.367 | 0.023 | 0.524 |
| Infliximab |  |  | 0.010 | 0.235 | **0.019** | **0.047** | 0.019 | 0.94 |
| Smoking (current) |  |  | 0.019 | 0.331 | 0.03 | 0.113 | 0.024 | 0.359 |
| Smoking (ever) |  |  | 0.018 | 0.634 | 0.031 | 0.419 | 0.023 | 0.49 |
| IBDsurgery |  |  | **0.011** | **0.021** | **0.020** | **0.028** | 0.024 | 0.225 |
| Ileocaecalresection |  |  | **0.011** | **0.029** | **0.020** | **0.023** | NA | NA |
| Colectomy with IPAA | | | **0.011** | **0.041** |  |  | **0.025** | **0.048** |
| L_location |  |  |  |  | 0.053 | 0.236 |  |  |
| B_behavior |  |  |  |  | 0.051 | 0.237 |  |  |
| HBI |  |  |  |  | 0.015 | 0.471 |  |  |
| E_extentofuc |  |  |  |  |  |  | 0.115 | 0.711 |
| SCCAI |  |  |  |  |  |  | 0.020 | 0.917 |
